# Land Use/Land Cover Change and Its Driving Forces in Shenkolla Watershed, South Central Ethiopia

**DOI:** 10.1155/2021/9470918

**Published:** 2021-02-18

**Authors:** Belayneh Bufebo, Eyasu Elias

**Affiliations:** ^1^Department of Natural Resource Management, Wachemo University, P.O. Box 667, Hosanna, Ethiopia; ^2^Center for Environmental Science, College of Natural and Computational Sciences, Addis Ababa University, Addis Ababa, Ethiopia

## Abstract

Land use change is one of the challenges that aggravate environmental problems. Understanding the scope of land use change, driving forces, and consequences is very crucial for proper management of land resources. We investigated land use/land cover changes using remote sensing data (for the years 1973, 1995, and 2017), and field observation, household survey, key informant interview, and focus group discussion were used to determine the drivers and consequences of land use/land cover changes in Shenkolla watershed, south central Ethiopia. Unsupervised and supervised classification techniques were employed to get thematic information from satellite imagery. ArcGIS 10.3 and QGIS v 3.0 softwares were used to accomplish the analysis. The results disclosed that Shenkolla watershed has changed significantly during the past 4 decades between 1973 and 2017. This observed change indicates a reduction in forest land and an increase in agricultural land. Forest land was reduced from 29.51% in 1973 to 20.52% in 2017, but agricultural land was expanded from 70.49% in 1973 to 79.48% in 2017. Agricultural expansion, policy change and social unrest, population pressure, shortage of farm land, and biophysical factors were major driving forces of the LU/LC changes. Environmental implications such as climate change, biodiversity loss, scarcity of basic forest products, habitat alteration, decline in quality and availability of water, and crop yield reduction are the consequences of the LU/LC change. The expansion of agricultural land at the expense of forest cover in Shenkolla watershed has negative implications on the natural resources and the livelihood of local people. Hence, appropriate measures need to be employed to reduce the dramatic change in land use and to harmonize environmental conservation with human livelihood.

## 1. Introduction

The complex and dynamic land use/land cover change at various scales has environmental implications [1, 2]. The main driving forces of LU/LC change can be traced to the consumption demands of the increasing population that is a major issue of concern in relation to change in the natural environment [3–5]. Land use change can trigger soil degradation and soil erosion, which changes watershed properties that may cause flooding in nearby areas [6]. Desertification, loss of biodiversity, habitat destruction, soil degradation, and a reduced ability of the watershed to sustain natural resources and ecosystem services are the consequences of land use/land cover change [7–9]. The consequences of land use change challenge conservation, management, and rehabilitation activities [10]. The relationship between land use/land cover change and its driving factors is complicated and dynamic. Some of the previous studies suggest that demographic changes contribute more than any other causative factors of land use/land cover changes [11]. Other studies suggest economic factors to be the major drivers of LU/LC change [12].

Over the past few decades, considerable LU/LC change has been happening in the highlands of Ethiopia. Previous studies indicated that the decrease of forest cover and expansion of agricultural land into steep slope areas not suitable for cultivation are significant forms of LU/LC change in most highlands of Ethiopia [13–15]. There was a substantial increase in cultivated land at the expense of forest cover in northwest [13], northeastern [16], and eastern [17, 18] Ethiopia. The loss of natural vegetation from 1973 to 2000 in Abijata Shala National Park and Zway-Awasa Basin was 83.4 and 70.1%, respectively [19]. On the other hand, some studies revealed the improvement of forest cover due to afforestation and land rehabilitation activities carried out by the community [20, 21]. Most previous studies on LU/LC changes were concentrated in specific areas, mainly in the Northern highlands and some areas in the rift valley lake basin of Ethiopia and quantified only the extent of land use/land cover changes using remote sensing images; however, they did not provide explanations on local people perception of driving forces of LU/LC change and associated consequences [22]. In addition, studies on land use/land cover changes are still very limited in the Omo Gibe river basin of south central Ethiopia. In order to implement measures to minimize LU/LC changes, it is important to understand how the community perceives the causes and consequences of LU/LC changes [23].

LU/LC change is more recognized as an important driver of environmental degradation and loss of quality on spatial and temporal scales. LU/LC change contributes significantly to climate change, reduction in forest cover, and biodiversity loss [24]. In addition, LU/LC change is one of the factors that influences runoff, soil loss, and stream flow [20]. Remote sensing data analysis has a limitation to explain nonspatial data such as derivers and consequences of land use/land cover change. As LU/LC change increases, linking information obtained through Earth observation with human perception is significant in gaining a comprehensive understanding of pattern of LU/LC changes, driving forces, and consequences [22, 25]. The study area, Shenkolla, is known to be productive in cereal production, but the area is exposed to sever erosion and soil loss triggered by LU/LC change. Hence, a thorough understanding of the extent of LU/LC change, driving forces, and consequences of LU/LC change is crucial to design more effective environmental policies and appropriate land management strategies for the entire watershed and beyond [26]. However, land use/land cover change of Shenkolla watershed is not investigated so far, as the result, the extent of such change, its driving forces, and consequences are poorly understood. Therefore, the main objective of the study was to analyze the LU/LC changes from 1973 to 2017 and its driving forces and consequences and to evaluate the coherence of community perception to the changes observed through the interpretation of remote sensing images in the study watershed.

## 2. Materials and Methods

### 2.1. Description of the Study Area

**Figure 1:**
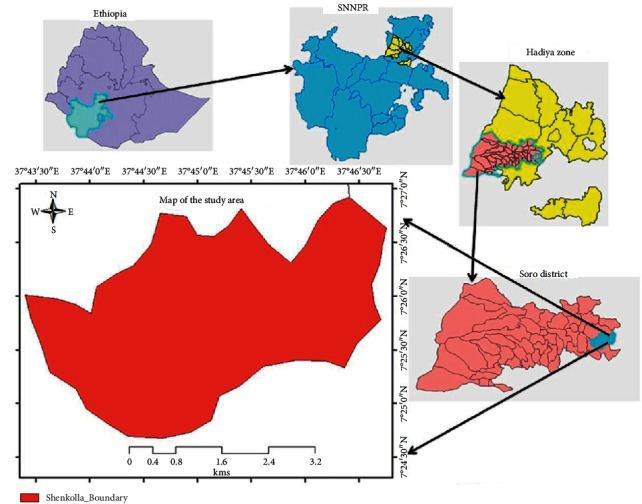
Map of the study site in southern Ethiopia.

The study was conducted in Shenkolla watershed, covering 1457 ha in south central Ethiopia. The geographical location of the area falls within the coordinates of 7°24′30″–7°27′0″ N latitude and 37°43′30″–37°46′30″ E longitude (Figure 1). The altitude ranges from 2200–2830 m, which is characterized by gently sloping to rolling plateaus with moderate to high relief hills and dissected side slopes.

**Figure 2:**
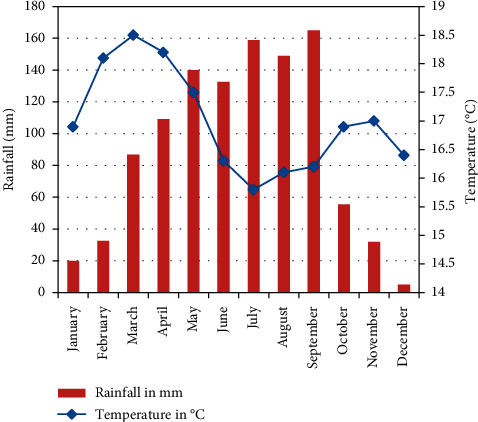
Mean monthly rainfall and temperature values of the study area.

The climate of the watershed is characterized generally as a tepid submoist midhighland with a long-term average rainfall of about 1107 mm with bimodal pattern having Belg (traditional division of the year with light rain) usually from March to May and the Meher (traditional division of the year with heavy rain) from June to September. The annual average temperature of the study area is 17.2°C (Figure 2).

Geological formation is dominated by the quaternary volcanics composed of acidic parent materials (rhyolites, trachytes, etc) [27]. Nitisols are the most dominant soil types along with Vertisols, Cambisols, and Planosols that cover extensive areas of agricultural fields [27].

Subsistence farming is the major source of livelihood relying on rain-fed crop cultivation with the major crops being wheat (*Triticum aestivum* L.), teff (*Eragrostis tef*) (Zucc. Trotter), maize (*Zea mays* L.), barley (*Hordium vulgare* L.), sorghum (*Sorghum bicolor* L.), horse beans (*Vicia faba* L.), and potato (*Solanum tuberosum* L.). The homestead garden fields are characterized by Enset (*Ensete ventricosum*) and with interspersed trees that increase the fertility of the soil. Under normal climatic conditions, the cultivation of crops is possible during both Belg (traditional division of the year with light rain) and Meher (traditional division of the year with heavy rain).

Due to the intense pressure of population growth and land scarcity, it is almost impossible for farmers to practice the appropriate fallow length. This resulted in dramatic land use/land cover changes in the watershed. The current land use/land cover types of the study area were categorized broadly into two categories: forest land and agricultural land. The forest cover of the study area also comprises natural and plantation forests, while the agricultural land includes cultivated land, small plots of grazing lands, and scattered rural settlements. During the past four decades, the conversion of forest into agricultural land in the Shenkolla watershed was quite intense. As a result, the agricultural land use class covers the largest proportion of the study area.

### 2.2. Data Sources

The main data sources for this research were semistructured questionnaires, complemented with field observations, remote sensing imagery, topographic maps, supportive data such as GPS records, and related literatures. This study involved the use of mixed methods to complement each other since the methods have their own weaknesses and strengths. Primary data were collected through extensive field observation, key informant interview, and focus group discussions. Ancillary data (training sites and ground control points) consisting of different LU/LC features and their location points were recorded using a Global Positioning System (GPS) instrument [28]. The images were freely obtained (downloaded) from the USGS Glovis website (http://glovis.usgs.gov/). ArcGIS 10.3 and QGIS v 3.0 software were used to classify the image and to delineate the study area. The acquisition dates, sensor path/row resolution, and the source of the images used in this study are summarized in Table 1.

### 2.3. Image Preprocessing and Classification Methods

All satellite images were geometrically corrected to the Universal Transfer Mercator coordinate system and georeferenced to the data in which Ethiopia has selected by the WGS (World Geodetic System) (zone 84). Moreover, preprocessing activity such as radiometric correction and a false color grid composite image are developed before classifying the images [29, 30].

Image classification was carried out by sorting pixels into a finite number of individual categories of data based on their data file values [31–33]. All pixels in an image were placed into LU/LC classes to draw out useful thematic information [34]. First, unsupervised classification was used to get the major land parcels, which then used for supervised classification. A total of 150 training sites were selected based on image interpretation keys during the field survey and from interviews with the local inhabitants. Reference points in different land use/land cover types were randomly recorded during the field survey using a hand-held Global Position System (GPS) for the 2017 images, the same as the procedure followed by [35, 36]. Supervised classification with maximum likelihood algorithm was used to classify the individual images independently using the ground control points collected from each LU/LC category [37, 38]. ArcGIS 10.3 and QGIS v 3.0 software were used for overall image processing. The way of classification of this study was adopted in such a way that it suits the purpose of the study. Finally, two land use/land cover classes were identified using independent classification of individual images from different dates for the same geographic location. These include agricultural land and forestland. The dispersed rural settlement and small scattered plots of grazing land were categorized as agricultural land use class. Land use/land cover classes of Shenkolla watershed and the corresponding description are displayed in Table 2.

### 2.4. Classification Accuracy Assessment

To perform accuracy assessment for the classified images, 100 random sample points (50 for each land use) in Arc GIS 10.3 were created for LU/LC mapping for the years 1973, 1995, and 2017, respectively. Ground control points recorded by using a hand-held GPS were used as the reference data to evaluate the results. In addition, reference points collected from the topographic map of 1973 and visual interpretation of the raw Landsat TM 1995 images as well as the personal knowledge of the study area and Google Earth images were used. The classification accuracy assessments of the resulting LU/LC layers were performed by examining the sample LU/LC class of the classified layer and the reference layer to discover similarities and differences. This means, the classified images were compared with the reference images by creating an error matrix [38]. By comparing the datasets, the proportion of pixels correctly classified was estimated. Error matrices were plotted as cross tabulations of the classified data versus the reference data and were used to assess the classification accuracy. Afterwards, the overall accuracy, user's and producer's accuracies, and the kappa coefficient were then derived from the error matrices. Overall accuracy was calculated using the following formula [39], as shown in equation (1), while the Kappa coefficient was calculated using the formula [40] shown in equation (2).

(1) $$\begin{array}{c} A=\frac{x}{y}*100, \end{array}$$

where *A* is the overall accuracy, *x* is the number of correct values in the diagonals of the matrix, and *y* is the total number of values of a reference point.

The Kappa coefficient is a measure of overall agreement of a matrix. The Kappa coefficient takes also nondiagonal elements into account [41]. The Kappa coefficient, which measures the difference between the actual agreement of classified map and chance agreement of random classifier compared to reference data, was calculated as follows:

(2) $$\begin{array}{c} K=\frac{N\sum_{i=1}^{r}xii-\sum_{i=1}^{r}\left(xi+*\;x\;+\;i\right)}{N^{2}-\sum_{i=1}^{r}\left(xi+*\;x\;+\;i\right)}, \end{array}$$

where *K* is the Kappa coefficient, *r* is the number of rows in the matrix, *xii* is the number of observations in row *i* and column *i*, *xi* *+* are the marginal totals of row *i*, *x* + *i* are the marginal totals of column *i*, and *N* is the total number of observations.

### 2.5. LU/LC Change Detection

The pattern of changes in terms of hectares for land use/land cover classes was computed for each mentioned time period and the extent of alteration in land use types within and between time periods was compared. The rate of change in hectares per year [42] and percentage share [43] of each land use class were computed to demonstrate the magnitude of the changes experienced between the periods using the following equations:

(3) $$\begin{array}{c} CA\left(\%\right)=\left(\frac{X_{2}-X_{1}}{X_{1}}\right)*100, \end{array}$$

(4) $$\begin{array}{c} \text{Rate of change}\left(\frac{\text{ha}}{\text{year}}\right)=\left(\frac{X_{2}-X_{1}}{y}\right), \end{array}$$

where *CA* (%) = percentage change in the area of land use and land cover type between initial time *X*_1_ and final time *X*_2_. *X*_1_ = area of land use/land cover type at the initial year. *X*_2_ = area of land use/land cover type at the final year. *Y* = time interval between the final and initial years.

### 2.6. Exploring the Drivers and Consequences of LU/LC Changes

**Figure 3:**
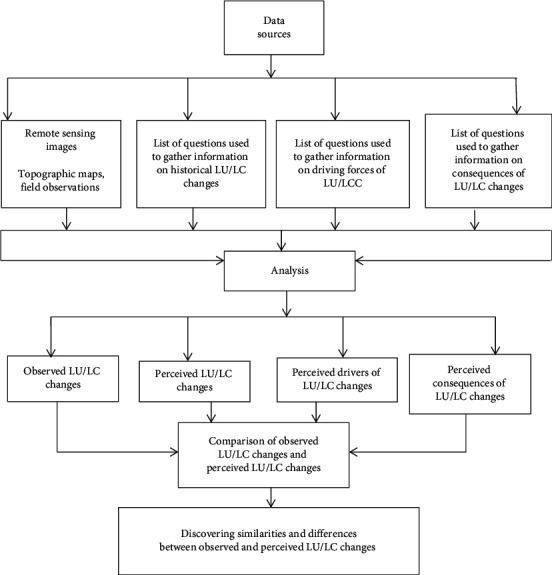
Methodology of comparison between observed and perceived LU/LC changes.

Household survey, key informant interviews, and focus group discussions were conducted to prove the correctness of the classified images and further come to know the possible major driving factors, and consequences of land use change in the watershed. A questionnaire with semistructured questions was used to assess the perception of local people on LU/LC change, its drivers, and consequences. The most appropriate age was decided to be 55 and above. Therefore, 100 respondents with age 55 and above were purposively selected to identify LU/LC changes, driving forces and consequences. Respondents were requested to explain how they perceived LU/LC dynamics in the watershed in different time periods assessed in this study. They evaluated the status of the land use/land cover change, its drivers, and consequences. Finally, the driving forces, consequences, and the direction of land use change were identified. Subsequently, the perceived LU/LC changes were compared with the land use/land cover changes observed from the remote sensing images interpretation (Figure 3).

## 3. Results and Discussion

### 3.1. Classification Accuracy Assessment

The reliability and accuracy of the classification was measured using a confusion matrix. The confusion matrix worked out the overall accuracy, producer and user accuracy, and kappa statistics with mathematical precision. Correctly classified values are shown on diagonals of the matrix, while incorrectly classified values are away from the diagonals. The overall accuracy for the classified images of the 1973, 1995, and 2017 was 85, 83, and 87%, respectively (Table 3). The kappa statistic for 1973, 1995, and 2017 LU/LC maps was 0.70, 0.66, and 0.74, respectively, showing a good level of agreement between the classified images and the referenced data. This image‐processing approach was found to be effective in producing compatible data of LU/LC changes. The report of the overall accuracy and accuracy of the individual groups of the three classified images is presented in Table 3.

### 3.2. Land Use/Land Cover Change Analysis

The land use/land cover change analysis showed that the study area has exposed to a marked land use change over the past four decades. LU/LC change detection between 1973, 1995 and 2017 of the study area indicated that there were significant conversions from forest land use to agricultural land. The loss of forest cover has been the most visible evidence of land use/land cover change in the Shenkolla watershed for the last 40 years. The land use/land cover classes of the study area were classified in to two classes, namely, forest land and agricultural land. The change detection statistics for four decades of the study area are presented in Table 4.

**Figure 4:**
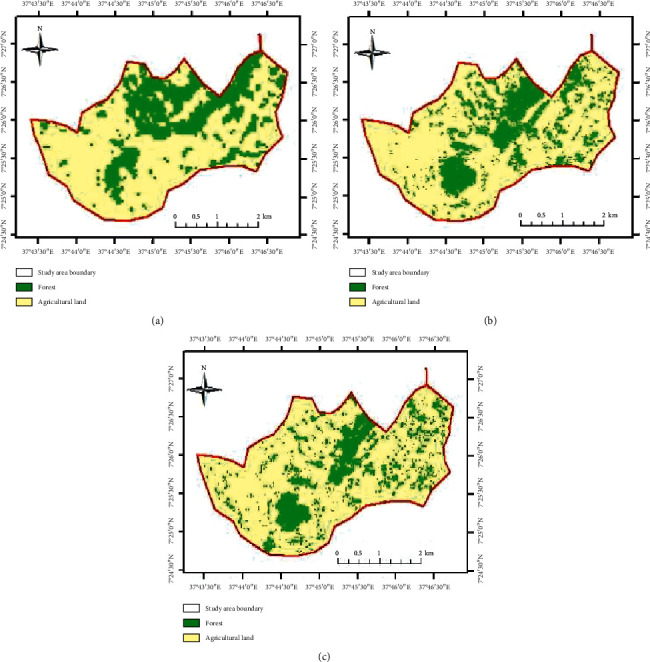
Land use/land cover changes of Shenkolla in the years (a) 1973, (b) 1995, and (c) 2017.

According to the produced LU/LC map (Figure 4), it was found that agricultural land was the dominant type of LU/LC class for the years 1973 and 1995 and 2017. The highest expansion of agricultural land at the expense of forest land was recorded in 1995. The map of the years 1973, 1995, and 2017 showing change in land use/land cover through over time due to various causes is presented in Figure 4.

### 3.3. Land Use/Land Cover Conversions between 1973 and 1995

The rate and trend of land use/land cover transformations varied to a significant degree between the time intervals under investigation. Land use/land cover changes are dynamic and nonlinear, that is, the conversion from forest land use class to agricultural land does not follow a similar pattern of change in different periods [44]. Change detection results of the first period (1973–1995) showed an increasing trend of agricultural land; on the contrary, forest land showed a decreasing trend. In 1973, there were 430 ha (29.51%) and 1027 ha (70.49%) of forest and agricultural land, respectively. However, forest land decreased from 430 ha (29.51%) to 346 ha (23.75%). This indicated that 5.76% of forest land has been converted to agricultural land use class and, as a result, the area coverage of the agricultural land was increased in the year 1995 (Table 4). These changes in land use/land cover systems have important environmental consequences through their impacts on soil, water, biodiversity, and microclimate [7]. The annual rate of change in forest land from 1973 to 1995 was −19.5%, but agricultural land increased annually by 8.2% (Table 5). The negative change in the forest area implies a decline in the area coverage of forest, whereas agricultural land was positive suggesting increasing area extent. Agricultural land gained from forest land; as the result, there was a significant loss of forest land in the watershed (Table 6). This result is in agreement with reports elsewhere in the Ethiopian highlands that showed agricultural land expansion at the expense of forest and grazing lands [45].

### 3.4. Land Use/Land Cover Conversions between 1995 and 2017

In the second period (1995 to 2017), the extent of forest land decreased from 346 ha (23.75%) to 299 ha (20.52%) and agricultural land increased from 1111 ha (76.25%) to 1158 ha (79.48%) (Table 4). This showed that agricultural land increased with the expense of forest land in the study area. In this period, 47 ha of forest land was changed into agricultural land in 22 years. This showed that agricultural land gained from forest land (Table 7). As a result, the area coverage of agricultural land class in the study area was increased by 47 hectares. During the second period, from 1995 to 2017, the annual rate of change in the area of forest land and agricultural land showed a decreasing and increasing trend by −2.14% and +2.14%, respectively. The decreasing trend of forest land is associated with the expansion of agricultural land to meet the food demands of the growing population. The negative and positive changes in change detection correspond to the gain or loss of that particular land cover.

### 3.5. Land Use/Land Cover Conversions between 1973 and 2017

Over the whole period of investigation (1973–2017), agricultural land increased from 70.49%–79.48%. On the other hand, forest land decreased from 29.51%–20.52% (Table 4). Generally, within these 44 years, 131 ha of forest land were changed into agricultural land. During this period, the annual rate of change of forest and agricultural land was −2.98% and +2.98%, respectively (Table 5). Agricultural land gained from forest land, as a result there was a significant loss of forest land in the watershed (Table 8). This shows that agricultural land was increasing significantly whereas forest land was shrinking in Shenkolla watershed.

### 3.6. The Transition Matrix

Evidences from this study showed that a substantial portion of the Shenkolla watershed undergoes great changes in land use/land cover. Agricultural expansion, the most prominent phenomenon, is most associated with the decline in forest lands. This is possibly a similar trend in that most studies pointed out the expansion of agricultural land to be at the expense of forestland in most areas in the Ethiopian highlands [15]. The findings of this study also visualized the most improperly used forest land use class needs urgent protection and conservation interventions. Information from this study on a significant reduction of forest land over time is important for land use planners and policy makers to take any intervention actions toward forest conservation. Moreover, the transition matrix can aid to know the altered land use due to conversion and helps to design good implementation strategies and to make a good decision for better management.

Generally, the results of the image classification are consistent with the findings of previous research conducted in different parts of the country. For example, the increase in cropland and decline in woodland were also observed in [1, 46].

### 3.7. Perception of LU/LC Change

Inhabitants interviewed were the heads of the household and with ages of 55 years and above, so that all interviewees have lived through the complete study period and were able to answer questions about all periods. All the interviewees correctly mentioned that currently forest cover of the study area has been declined as compared to the beginning of the study period. This suggests that the respondents generally had a good perception of the historical land cover pattern of the study area. Throughout the study period, the high increase in agricultural land at the expense of forest cover was perceived correctly by all respondents in the 1973–2017 period. The overall perception of the respondents on the decline of forest cover as the result of agricultural expansion was consistent with the LU/LC change observed in the remote sensing data interpretation.

### 3.8. Driving Forces of LU/LC Change in the Shenkolla Watershed

Understanding the causes and consequences of deforestation is critical to researchers, policy makers, and land managers because it helps to take appropriate measures [47]. Interviewees have indicated that agricultural expansion was identified as proximate causes of deforestation. Population increase, biophysical factors, policy changes, and social unrest were identified as underlying forces that led to occurrence of proximity causes of deforestation (LU/LC change) in the study area. According to the Ethiopian Central Statistics Report [48], the population of the study area has been increased at an alarming rate in the past four decades, leading to a similar increase in high demand for foodstuffs from agricultural expansion.

**Figure 5:**
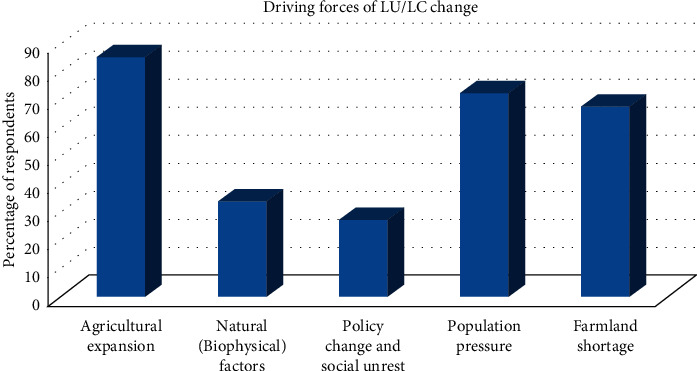
Driving forces of land use change based on community perception.

As clearly indicated in the LU/LC change analysis, agricultural land showed significant increase in the Shenkolla watershed over the last 44 year period (1973–2017). Similarly, a significant number of respondents (85.4%) indicated that human interference mainly agricultural expansion was the main causes of land use/land cover change (Figure 5). Substantial increase in demand for food has resulted in an expansion of agricultural lands by encroaching on uncultivated areas of forest lands.

Increase in population has implications for land resources as the need to produce food and the demand for settlement and fuel wood increase in response to growing population needs. In other words, the shortage of farm land stimulated by population growth forced local community to clear forest on steep slopes, which aggravated erosion problem and soil fertility decline. Rapid population growth of the study area resulted in expansion of a farmland and threatened the land covered with forest. Fast population growth and the consequent high pressure on resources are expected to have an adverse effect on the existing natural resources of the area. Household survey, focus group discussion, and key informant interview confirmed that population growth is an important indirect driver of land use/land cover change. Accordingly, 72.8% of the respondents pointed out high population pressure as the driver of land use/land cover change. Moreover, the shortage of farmland triggered by population growth is perceived as a driver of land use change by 68% of the respondents (Figure 5).

The ownership of all lands by the state in the whole country during the 1974–1991 led to a lack of sense of belongingness to natural resources by the individual farmers, which in turn triggered significant deforestation and agricultural expansion [49]. Moreover, the information obtained from key informants and focus group discussions revealed that there was a high conversion of forest cover to agricultural land, especially during periods of social unrest and regime change, to meet the demands of the growing family size at each household level. Failure of institutions to deliver its responsibility and law enforcement led to high deforestation and agricultural land expansion. Based on the analysis of the response of the questionnaire survey, 27.6% of the total respondents revealed that the regime change accompanied with social unrest as the indirect driver of land use change (Figure 5).

Biophysical (natural) factors as drivers of LU/LC change were mentioned most often in relation to anthropogenic drivers. For example, soil degradation is most often linked to anthropogenic activities (intensive cultivation and inadequate soil management) and poor natural conditions, such as the sloping nature of landscapes aggravates soil erosion that result in soil fertility decline. Climate variability was explained in relation to weather extremes. Generally, 34% of the respondents perceived biophysical or natural factors (topography, climate change, and soil type) as direct drivers of land use/land cover change (Figure 5). Biophysical factors such as soils, rainfall variability, and prolonged drought also have an impact on land use changes. Soils vary in their resistance to erosion partly based on texture and amount of organic matter. On steep slopes, soils are generally shallower and their nutrient and water storage capacities are limited. Drought and floods are two important climatic events responsible for soil chemical and physical degradation. The biophysical factors may act as constraints to agriculture production as they offer certain kinds of limitations to production. Through focus group discussion and key informant interviews, it was revealed that the deterioration of soil fertility with continuous cultivation and climate extremes consequently declined agricultural productivity. As the result, the local people seek extra land by clearing land covered by forest as opposed to increasing production and productivity on existing areas of agricultural land.

In general, many driving forces were responsible for the observed changes in land use; however, agricultural expansion, farmland shortage that has a direct relation with population pressure; land tenure policy and social unrest during the transition period; and natural (biophysical) factors were identified by respondents as the driving forces of land use change in Shenkolla watershed. The respondents' perception of drivers of LU/LC change was in agreement with the findings of previously conducted research in different parts of the country, for example, population pressure [50], deforestation, agricultural expansion, lack of alternative livelihoods, and land policy [1].

### 3.9. Consequences of LU/LC Change in the Shenkolla Watershed

Land use/land cover changes have wide range of consequences at all spatial and temporal scales. Because of these effects, LU/LC change has become one of the major problems for environmental change as well as natural resource management. The conversion of forest cover into agricultural land of the study area has great ecological consequences. Respondents have mentioned climate change, biodiversity loss, scarcity of basic forest products, habitat alteration leading to human-wildlife conflicts, decline in quality and availability of water, reduction in crop yield as a result of accelerated runoff, and soil fertility decline to be the major consequences of land use/land cover change in the study watershed.

**Figure 6:**
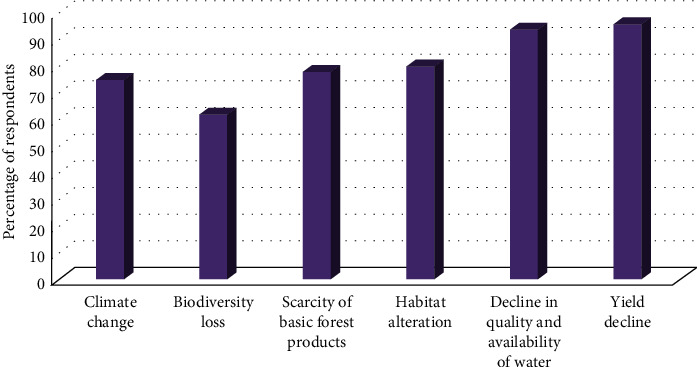
Consequences of LU/LC change based on perceptions of respondents.

Majority of the respondents (75%) mentioned that the local climate change (erratic rain and drought) is caused by the ongoing land use/land cover change (Figure 6). Moreover, the interviewees indicated that LU/LC change and associated climate change over time directly affects the livelihood of the subsistent farmers by affecting crop production, since most of them are completely dependent on rain-fed agriculture.

Another consequence of the LU/LC change was the loss of biodiversity. Changes in environmental conditions and natural setting of the land and its cover greatly affected the life cycle and the survival of various plants and animals. More than 62% of the respondents said that some species of plants and animals previously found in the study area disappeared mainly as a result of unregulated deforestation and agricultural expansion (Figure 6). The respondents believed that the diversity of both plants and animals was declined in the study watershed.

Forest products are very essential in the daily life of the inhabitants of Shenkolla watershed, since most of them depend on forest products for construction, cooking, heating, and light. However, continuous deforestation led to the scarcity of forest products. More than 78% of the respondents mentioned the occurrence of scarcity of forest products in the watershed (Figure 6).

Land cover (vegetation cover) highly controls the runoff. Land use/land cover change can influence soil chemical and physical properties because of different anthropogenic activities, namely, deforestation and agricultural expansion associated with intensive cultivation. More than 96% of the respondent farmers have also perceived that crop yield has been declined due to accelerated runoff, soil fertility decline, and erratic rain, which is mainly caused by the change in LU/LC (deforestation) (Figure 6).

LU/LC change was also mentioned as a cause of decline in quality and availability of water. A majority of the respondents indicated that LU/LC change from forest to intensively cultivated land increased the overall immediate surface runoff and sediment concentration in rivers. More than 94% of the interviewed farmers indicated that drying up of springs and decline in river water quality and quantity are a major problem caused by land use change (unsustainable land management practices) (Figure 6). They quoted the amount of water in the rivers, seasonality of the springs, changes in rainfall patterns, distances to water collection points, and depth of water wells as indicators of changes in water quantity. Water scarcity was most prevalent during the dry months of the year (December to March).

LU/LC change (deforestation) by altering habitat greatly affected the wildlife in Shenkolla watershed. The results of descriptive statistics indicate that the respondents were aware about the effects of habitat alteration on wildlife. More than 80% of the respondents said as a result of habitat destruction, some species of wild animals and birds previously found in the study area disappeared (Figure 6).

### 3.10. Visual Indicators of Soil Degradation Caused by LU/LC Change

**Figure 7:**
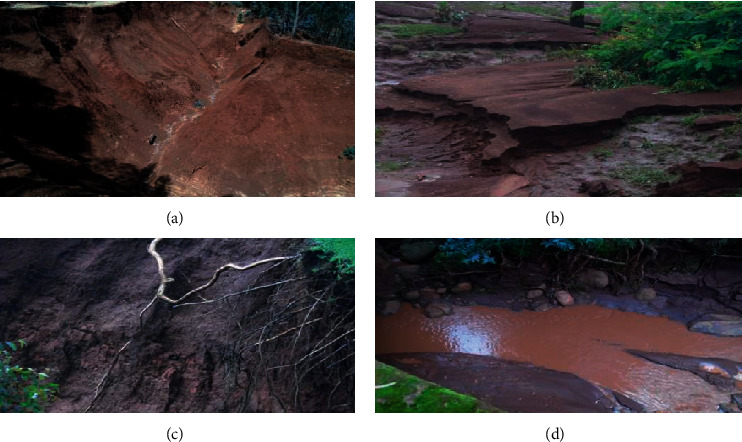
(a) Deep gully, (b) sediment at the bottom slope, (c) tree root exposure, and (d) river water polluted with eroded soil in Shenkolla watershed (source: photo taken during field work, 2017).

One of the adverse effects of land use/land cover change in the study watershed was soil degradation. Some of the observed visual indicators of soil degradation (loss) in the study area were intensive erosion, land slide, deep gully formation, river water pollution, tree root exposure, and the piling up of sediment (Figure 7).

Other notable indicators of soil degradation in the study area include stunted crop growth which results in yield decline and stones on the surface of cultivated lands making plough difficult. As a result, subsistence farming and smallholder agriculture that is most common in the study watershed is less productive in terms of yield per unit area of land. Similarly, in [51, 52], it was explained that rapid expansion of agricultural land into a steeper slopes and destruction of vegetation cover have aggravated soil erosion and degradation in the highlands of Ethiopia which resulted in the depletion of fertile soil.

Land degradation resulted from LU/LC change restricts people from accessing important ecosystem services, influences the livelihoods of the people, and increases the risk of poverty. The vulnerability of people that depend on land can be determined by their sustainable use of land and the effectiveness of their attempt to address land degradation through sustainable land management practices. Success in fighting land degradation requires an improved understanding of its causes and severity of consequences. Thus, our study provided a complete picture of the watershed through complementing remote sensing data with qualitative data collected from a local community through interview and discussion. This study suggests that maintaining sustainable use of natural resource and promoting sustainable agriculture will only be achieved when the perceptions of local people are understood well in order to act accordingly. The local people deserve to be supported to promote sustainable land management practices that is used to their environment and socioecological context since they have an important role and responsibility as stewards of land.

## 4. Conclusions

Analysis of remote sensing data revealed that a remarkable decline in forest cover and a significant expansion of agricultural land in Shenkolla watershed during the past 4 decades. The extent of forest land was reduced from 29.51% in 1973 to 20.52% in 2017. Agricultural land was expanded from 70.49% in 1973 to 79.48% in 2017. This shows that agricultural land increased at the expense of forest land. The direction of LU/LC changes perceived by the respondents was consistent with the result obtained from remote sensing image interpretation. Agricultural expansion, policy change and social unrest, population pressure, shortage of farm land, and biophysical factors were the major driving forces of the LU/LC changes. Environmental implications such as climate change, biodiversity loss, scarcity of basic forest products, habitat alteration, decline in quality and availability of water, and crop yield reduction are resulted from the LU/LC change. If this tendency of LU/LC change continued, it will have serious environmental and economic consequences with impact on livelihood of local people. Thus, appropriate measures that ensure wise use of natural resources and efficient utilization of land are very much critical.

## Figures and Tables

**Table 1:** Characteristics of images used for land use/land cover change analysis.

Satellite image	Sensor	Path/row	Resolution (m)	Bands used	Acquisition date	Source
Landsat 1	MSS	181/55	57 ∗ 57	1, 2, 3 and 4	31/01/1973	USGS
Landsat 5	TM	169/55	30 ∗ 30	1, 2, 3, 4, 5 and 7	21/01/1995	USGS
Landsat 8	OLI_TIRS	169/55	30 ∗ 30	1, 2, 3, 4, 5, 6 and 7	2/02/2017	USGS

**Table 2:** Descriptions of land use/land cover types for the period 1973–2017.

Land use/land cover class	Description
Forest land	Land covered with natural and plantation forests
Agricultural land	Areas of land which include cultivated outfields (areas of land used for growing various crops), homestead garden fields, rural settlements, and small scattered plots of grazing lands

**Table 3:** Accuracy assessment (in percent) of the 1973, 1995, and 2017 LU/LC maps.

Land use/land cover	1973		1995		2017	
	Accuracy (%)			
	User's	Producer's	User's	Producer's	User's	Producer's
Agricultural land	88	83	86	81	90	85
Forest land	82	87	80	85	84	89
Overall accuracy (%)	85		83		87	
Kappa coefficient	0.70		0.66		0.74	

**Table 4:** Areas and percentages of LU/LC classes for the years 1973, 1995, and 2017.

LU/LC category	1973		1995		2017	
	Area (ha)	%	Area (ha)	%	Area (ha)	%
Agricultural land	1027	70.49	1111	76.25	1158	79.48
Forest land	430	29.51	346	23.75	299	20.52
Total	1457	100	1457	100	1457	100

**Table 5:** Percent and rate of land use changes in the Shenkolla watershed from 1973 to 2017.

Land use/land cover	Percent change/year			Rate of change in ha/year		
	1973–1995	1995–2017	1973–2017	1973–1995	1995–2017	1973–2017
Agricultural land	8.2	4.2	12.8	+3.82	+2.14	+2.98
Forest	−19.5	−13.6	−30.5	−3.82	−2.14	−2.98

**Table 6:** Land use/land cover change matrix (ha) between 1973 and 1995.

1995				
	LU/LC	Agricultural land	Forest	Total
1973	Agricultural land	**861**	166	1027
	Forest	250	**180**	430
	Total	1111	346	1457

**Table 7:** Land use/land cover change matrix (ha) between 1995 and 2017.

2017				
	LU/LC	Agricultural land	Forest	Total
1995
	Agricultural land	**112**	99	1111
	Forest	146	**200**	346
	Total	1158	299	1457

**Table 8:** Land use/land cover change matrix (ha) between 1973 and 2017.

2017				
	LU/LC	Agricultural land	Forest	Total
1973
	Agricultural land	**852**	175	1027
	Forest	306	**124**	430
	Total	1158	299	1457

## Data Availability

The data used to support the findings of this study are available upon request from the corresponding author.
